# Preservation of Mouse Sperm by Convective Drying and Storing in 3-O-Methyl-D-Glucose

**DOI:** 10.1371/journal.pone.0029924

**Published:** 2012-01-17

**Authors:** Jie Liu, Gloria Y. Lee, Joel A. Lawitts, Mehmet Toner, John D. Biggers

**Affiliations:** 1 Center for Engineering in Medicine and Surgical Services, Massachusetts General Hospital, Harvard Medical School and Shriners Hospital for Children, Boston, Massachusetts, United States of America; 2 Beth Israel Deaconess Medical Center, Boston, Massachusetts, United States of America; 3 Department of Cell Biology, Harvard Medical School, Boston, Massachusetts, United States of America; Konkuk University, Republic of Korea

## Abstract

With the fast advancement in the genetics and bio-medical fields, the vast number of valuable transgenic and rare genetic mouse models need to be preserved. Preservation of mouse sperm by convective drying and subsequent storing at above freezing temperatures could dramatically reduce the cost and facilitate shipping. Mouse sperm were convectively dried under nitrogen gas in the Na-EGTA solution containing 100 mmol/L 3-O-methyl-D-glucose and stored in LiCl sorption jars (Relative Humidity, RH, 12%) at 4°C and 22°C for up to one year. The functionality of these sperm samples after storage was tested by intracytoplasmic injection into mouse oocytes. The percentages of blastocysts produced from sperm stored at 4°C for 1, 2, 3, 6, and 12 months were 62.6%, 53.4%, 39.6%, 33.3%, and 30.4%, respectively, while those stored at 22°C for 1, 2, and 3 months were 28.8%, 26.6%, and 12.2%, respectively. Transfer of 38 two- to four-cell embryos from sperm stored at 4°C for 1 year produced two live pups while 59 two- to four-cell embryos from sperm stored at 22°C for 3 months also produced two live pups. Although all the pups looked healthy at 3 weeks of age, normality of offspring produced using convectively dried sperm needs further investigation. The percentages of blastocyst from sperm stored in the higher relative humidity conditions of NaBr and MgCl_2_ jars and driest condition of P_2_O_5_ jars at 4°C and 22°C were all lower. A simple method of mouse sperm preservation is demonstrated. Three-O-methyl-D-glucose, a metabolically inactive derivative of glucose, offers significant protection for dried mouse sperm at above freezing temperatures without the need for poration of cell membrane.

## Introduction

Mice are a widely used model for genetic and medical research. Instead of maintaining mouse colonies, sperm preservation provides a reliable and efficient way of preserving specific genotypes. Cryopreservation and freeze drying has been used for sperm preservation [1], [2]. Cryopreservation requires ultra-low temperature storage conditions, e.g., in liquid nitrogen, which can be costly over time and is not convenient for sample shipment. Freeze drying affords storage at ambient temperature yet involves a long and complicated process and sophisticated equipment. In contrast, evaporative drying, involving drying under a stream of dry gas (e.g. nitrogen) at ambient temperature, is an alternative procedure for sperm preservation which is rapid and does not require elaborate equipment. The technique mimics a natural process used by many animals and plants in nature to survive extreme weather conditions (cold or dryness) by accumulating large amount of sugars, such as glucose, trehalose, and sucrose, in their cells. It is believed that sugars protect these organisms from freezing and dehydration damage by glass formation, direct interactions with membrane components and proteins, and providing an energy source [3]–[5]. Mouse spermatozoa have been protected against desiccation by the artificial introduction of trehalose, a non-permeable dissacharide, into the cells by porating them with alpha-hemolysin [6], [7].

Poration requires extra steps and may be avoided by using the alternative sugar – glucose. Although glucose is rapidly transported across the cell membrane into cells and has reasonable glass forming properties, it is unsuitable as a protectant in artificial desiccation systems because it is rapidly metabolized and is toxic at high concentrations necessary for stabilization in the dry state. Three-O-methyl-D-glucose (3-OMG) is also rapidly transported into cells [8], [9] and it accumulates because it is not metabolized. It has been used as a cryoprotectant for the cryopreservation of liver cells [4] and for improving desiccation tolerance of keratinocytes [10]. We now report that 3-OMG can protect convectively dried mouse sperm stored above freezing temperatures for at least one year.

## Materials and Methods

### Animal donors

Two to 4 months old B6C3F1 female mice and 3 to 9 months old B6D2F1 male mice (Jackson Laboratories, Bar Harbor, ME) were used as oocyte and sperm donors. The males were selected from those having been mated at least 1 week before using as sperm donors. All procedures involving animals have been reviewed and approved by the Massachusetts General Hospital Subcommittee on Research Animal Care (no. A3596-01).

### Reagents and media

The Na-EGTA solution used for sperm preparation was 10 mmol/L Tris-HCl buffer supplemented with 50 mmol/L each of NaCl and EGTA with pH adjusted to 8.2–8.4 [11]. FHM and KSOM ^AA^ were purchased from Millipore (Billerica, MA). All other reagents were purchased from Sigma-Aldrich (St. Louis, MO) unless otherwise indicated.

### Sperm sample preparation

Male mice were anesthetized with Isoflurane U.S.P. (Abbott laboratories, Chicago, IL) and then killed by cervical dislocation. For each experiment, the caudal epididymides were excised and placed in 1 ml EGTA solution. The epididymides were cut with a fine needle in several places to allow the escape of sperm into the EGTA solution for 10 min at 37°C. Then the sperm suspension was transferred into a 1.5 ml conical tube for the live sperm to swim up for 10–15 min at room temperature. A sperm suspension from the upper half of the column in the conical tube was used.

An aliquot of sperm suspension (200 µl) was added in 200 µl of 200 mmol/L 3-OMG in Na-EGTA solution in a round bottom 17×100 mm culture tube at room temperature and mixed well to allow transport of 3-OMG into the sperm for 30 min before cooling it in an ice bath [4]. The final concentration of 3-OMG was 100 mmol/L and the sperm concentration ranged from 5–10 million/ml depending on the male.

A mixture of ice cubes and water was placed in the bath of Cole-Parmer Ultrasonic Cleaner (Model 08849-00, Vernon Hills, IL) which was run for 20–30 min to cool. Without turning off the Ultrasonic Cleaner, all but a few smallest pieces of ice were removed from the bath just prior to use. Then, the culture tube containing 400 µl of 3-OMG sperm suspension or pure sperm suspension without 3-OMG was inserted 2/3 way down below the surface of the ice water bath and sonicated for 3–4 s. A small drop of the suspension was examined under a microscope to estimate percentage of sperm heads separated from tails. This process was repeated, if needed, until 60%–80% of the sperm heads had separated. The sperm head preparation was kept on ice during the drying process [12], [13].

The drying solutions consisting of 50, 100, 200, 400, 500, and 600 mmol/L of 3-OMG were prepared by dissolving different amount of 3-OMG in the Na-EGTA solution. The solutions were filter sterilized.

### Convective drying

**Figure 1:**
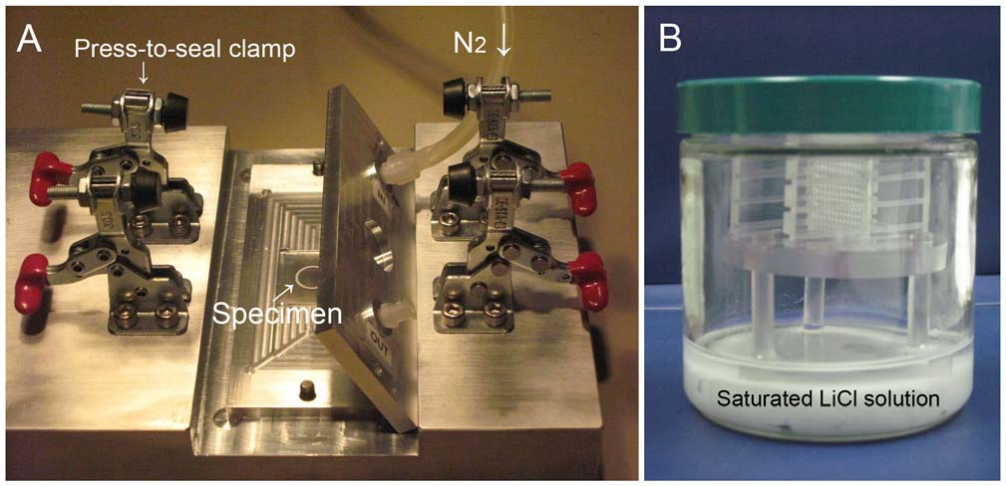
Drying chamber and storage jar. A) Drying chamber (Patent pending). It is made of aluminum and held together by two sets of press-to-seal clamps instead of 10 screws to provide increased ease of operation and a reliable system for long term reproducible drying process. The chamber is also designed to hold a 25 mm×25 mm square glass slide with a 10-mm diameter etched ring in the center of the slide. B) LiCl storage sorption jar. Each sorption jar contains a plastic system consisting of a lower tri-pot and a removable upper rack to accommodate eight 25 mm×25 mm glass slides.

The convective drying procedure was similar in principle and parameter to that described previously [14]. An improved drying chamber, made by the Quad Machine Shop (Harvard Medical School, Boston, MA), is made of aluminum and held together by two sets of press-to-seal clamps instead of 10 screws. It provides increased ease of operation and a reliable system for long term reproducible drying process (Figure 1A). The chamber is also designed to hold a 25 mm×25 mm square glass slide with a 10 mm diameter etched ring in the center of the slide (Gold Seal Products, Portsmouth, NH; custom-cut by Moliterno Inc., Pepperell, MA) over which a stream of ultra pure dry nitrogen gas flows at precisely controlled rate. Twenty microliter size sperm samples with 50, 100, 200, 400, 500, and 600 mmol/L of 3-OMG were dried by a stream of nitrogen gas at 10 l/min for 5, 6, and 7 min each, followed by baking in an oven at 85°C for 6–7 days to determine effects of the 3-OMG concentration and drying time on moisture content. Sperm samples with 100 mmol/L 3-OMG were dried for 6 min and stored in sorption jars containing NaBr, MgCl_2_, LiCl, or P_2_O_5_ at 4°C and 22°C. For each storage experiment, additional slides containing sperm samples were prepared and baked as moisture content controls. Slides were weighed before and after convective drying and after baking on an ultra microbalance (Mettler Toledo Inc., Columbus, OH) that has a readability of 0.1 µg. Moisture content was calculated as described [6]:
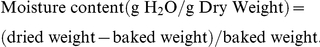



### Salt sorption storage jars

Saturated solutions of NaBr, MgCl_2_, and LiCl were prepared with varying salt to water ratios in 16 oz. wide mouth screw-top glass jars (Cole Parmer, Vernon Hills, IL) and allowed to equilibrate at 4°C and 22°C for 2 weeks before use [15], [16]. In addition, P_2_O_5_ powder without water was placed in a jar to create the driest condition. The relative humidity of each salt sorption jar was measured with an Omega hygrometer/thermometer (Model RH 200, Omega, Stamford, CT) custom fitted to the screw-top of the glass jars. Multiple readings of relative humidity and temperature were made over 2–3 weeks for each salt jar to determine humidity levels and stability at each temperature condition.

A custom designed plastic system consisting of a lower tri-pot and a removable upper rack to accommodate eight 25 mm×25 mm glass slides was made (Quad Machine shop, Dept. Systems Biology, Harvard Medical School, Boston, MA) to insert into each glass salt jar for sample storage (Figure 1B).

### Preparation of oocytes

Superovulation was induced in B6C3F1 by the intraperitoneal injection of 0.1 ml P.G. 600 (Intervet Inc., Millsboro, DE). This is equivalent to 5 IU serum gonadotropin (PMSG) and 2.5 IU human chorionic gonadotropin (HCG). A second injection followed 48 h later. Oocytes were retrieved 14 to 15 h after the second injection. Cumulus-oocyte complexes were released from the ampulla of the oviduct and incubated in 0.3 mg/ml hyaluronidase in FHM for 3–5 min to remove the cumulus cells. Denuded oocytes were washed and cultured in KSOM^AA^ medium at 37°C with 5% CO_2_ in humidified air until use.

### Intracytoplasmic sperm injection (ICSI)

ICSI using a conventional injection pipette was applied as described [17]. Briefly, each dried sperm sample was rehydrated by adding 20 µl of sterile distilled water. A few micro liters of the rehydrated sperm were mixed with 10% polyvinyl pyrrolidone (PVP). Injection pipettes with ∼5 µm inner diameter were used (custom made by Microjek, Fort Lauderdale, FL). A new drop of sperm in PVP was prepared after 15–20 oocytes were injected. Injected oocytes were left in the operation medium (FHM supplemented with 10% fetal bovine serum) at room temperature for 15–30 min before being transferred to KSOM^AA^ medium and cultured. Lysed oocytes were counted and removed from the culture.

### Embryo culture

Embryos were cultured in drops (50 µl/drop) of KSOM^AA^ medium [18] placed on a 60×15 mm suspension culture dish covered with mineral oil at 37°C with 5% CO_2_ in humidified air. Five to ten embryos were placed in each droplet. Numbers of two-cell embryos, compact morulae, and blastocysts were counted daily for 6 days.

### Embryo transfer

Embryos were transferred at the two- to four-cell stage (24–48 h post-ICSI) into day 0.5 pseudopregnant ICR females, which had been produced by natural mating with vasectomized males. The number of embryos transferred depended on the number of two- to four-cell embryos and the number of recipients available on a given day. Embryos were allowed to develop to term. Live births were allowed to mature to 3 weeks of age.

### Statistics and data analysis

Unordered, singly ordered, and doubly ordered contingency tables were analyzed using the exact Fisher, Kruskal-Wallis, and Jonckheere-Terpstra tests of significance, respectively. The computations were done using the StatXact 8 Package (Cytel Inc., Cambridge, MA). Logistic regression was applied using the LogXact 8 Package (Cytel Inc., Cambridge, MA). Differences were considered significant at P<0.05.

## Results

### Relative humidity levels of salt sorption jars

The relative humidity levels in salt sorption jars for NaBr, MgCl_2_, LiCl, and P_2_O_5_ at 4°C were 70%, 37%, 12% and 0%, respectively, and 61%, 35%, 12% and 0% at 22°C, respectively. There is little change in moisture contents of dried sperm samples dried in 100 mmol/L 3-OMG for 6 min and stored in LiCl jars for 1, 2, and 3 months at 4°C (+0.01, +0.04, and +0.02, respectively) and 22°C (−0.05, −0.05, and −0.04, respectively).

### Effects of 3-OMG concentration and drying time on moisture content

Moisture content ranged from 0.19 to 1.63 when samples with 50, 100, 200, 400, 500, and 600 mmol/L of 3-OMG were dried for 5, 6, or 7 min (Table 1). The corresponding glass transition temperatures for the 3-OMG samples were between −45.38°C and −62.99°C as calculated from the phase diagram of glucose-water system [19] (Table 2). When samples with 50 mmol/L and 100 mmol/L 3-OMG were dried for 6 or 7 min, moisture content fell into a range of 0.2 to 0.3 g H_2_O/g Dry Weight. Moisture content in this range offered better protection of dried sperm samples, assessed by the rate of blastocyte development after ICSI from our previous study [6]. Since sperm samples with 100 mmol/L of 3-OMG dried for 6 min reached the lowest moisture in the shortest time, this protocol was selected for the rest of the study.

**Table 1:** Moisture content of sperm samples in various concentrations of 3-OMG dried for 5, 6, and 7 minutes.

	3-OMG concentration (mmol/L)					
Drying time (min)	50 (n)	100 (n)	200 (n)	400 (n)	500 (n)	600 (n)
5	1.01 (6)	1.63 (6)	1.46 (3)	1.12 (3)	1.22 (6)	1.22 (3)
6	**0.28 (6)**	**0.23 (11)**	0.36 (3)	0.47 (3)	0.37 (6)	0.35 (3)
7	**0.19 (6)**	**0.21 (6)**	0.25 (3)	0.25 (3)	0.35 (6)	0.32 (3)

**Table 2:** Glass transition temperatures (°C) of 3-OMG corresponding to the moisture contents of the sperm samples.

	3-OMG concentration (mmol/L)					
Drying time (min)	50 (n)	100 (n)	200 (n)	400 (n)	500 (n)	600 (n)
5	−52.77	−62.99	−59.85	−54.41	−55.89	−55.89
6	**−45.88**	**−45.58**	−46.39	−47.20	−46.46	−46.32
7	**−45.38**	**−45.48**	−45.70	−45.70		

### Is 3-OMG needed to protect sperm from evaporative drying?

Sperm were dried in the Na-EGTA solution with and without 100 mmol/L 3-OMG and stored in LiCl jars at 4°C for 1 and 3 months. Thus the experiment compared four treatment groups (Table 3). The rates of fertilization (number of two-cells/number of oocytes survived injection) in the four groups were not significantly different (P = 0.384), although there was a suggestion that by 3 months storage without 3-OMG the rate had slightly declined (∼10%). Overall the fertilization rate was high [187/223 (83.9%)]. The developmental stages reached in the four groups after culture are shown in Table 4. The rates of blastocyst formation, after storage for 1 and 3 months, were significantly higher when 3-OMG was present in the medium compared to when it was absent (P<10^−5^ in both cases). A few blastocysts developed after 1 and 3 months storage when 3-OMG was absent, but most ceased to develop before the morula stage (Table 4).

**Table 3:** Fertilization rate using sperm dried in the Na-EGTA solution with and without 100 mmol/L 3-OMG and stored in LiCl jars at 4°C for 1 and 3 months.

Drying solution	Storage time (mon)	No. injected	No. survived (%)	No. 2-cell (%^a^)
3-OMG	1	118	62 (52.5)	54 (87.1)
no 3-OMG	1	104	45 (43.3)	40 (88.9)
3-OMG	3	87	54 (62.1)	45 (83.3)
no 3-OMG	3	110	62 (56.4)	48 (77.4)

a Percent of survived oocytes developed to two-cell embryos.

Probability that the fertilization rates are the same in all drying conditions = 0.0004 (Exact Fisher test).

**Table 4:** The distribution of embryos at various stages of development 5 days following ICSI using sperm dried with and without 3-OMG and stored at 4°C for 1 and 3 months.

		No. of embryos (%)					
Drying solution	Storage time (mon)	2-cell	3,4-cell	5–8-cell	Compac morulae	Blastocysts	Total embryos
3-OMG	1	2 (3.7)	1 (1.9)	2 (3.7)	17 (31.4)	32 (59.3)	54 (100)
no 3-OMG	1	0	12 (30)	7 (17.5)	12 (30)	9 (22.5)	40 (100)
3-OMG	3	1 (2.2)	4 (8.9)	4 (8.9)	17 (37.8)	19 (42.2)	45 (100)
no 3-OMG	3	2 (4.2)	25 (52.1)	11 (22.9)	5 (10.4)	5 (10.4)	48 (100)

Probability that the patterns of development are the same in all drying conditions <0.0004 (Exact Kruskal-Wallis Test).

### The effects of time and temperature when sperm were stored after being convectively dried with 3-OMG

#### Survival and fertilization

Mouse sperm were convectively dried in the Na-EGTA solution containing 100 mmol/L 3-OMG for 6 min and stored in LiCl jars at 4°C and 22°C for 1 to 12 months. When the samples stored at 4°C were used, 49.6%–58.6% of the oocytes survived injection, with an average of 54.0%. Of those survived injection, 82.8%–92.9% developed to two-cell embryos. When the sperm stored at 22°C were used, 49.6%–57.0% of the oocytes survived injection, with an average of 53.4%. Of those survived injection, 77.9%–91.2% developed to two-cell embryos. There was no difference among the percentages of survived oocytes developing to two-cell embryos when sperm stored for different period of time at 4°C (P = 0.276) or 22°C (P = 0.061) were used (Table 5).

**Table 5:** Number of oocytes survived injection and number of survived oocytes developed to two-cell embryos following ICSI using sperm dried with 100 mmol/L 3-OMG for 6 minutes and stored in LiCl jars for 1 to 12 months at 4°C and 22°C.

Temp-erature	Storage time (mon)	No. injected	No. survived (%)	No. 2-cell (%^a^)
4°C	1	193	106 (54.9)	91 (85.8)
	2	139	69 (49.6)	58 (84.1)
	3	126	64 (50.8)	53 (82.8)
	6	86	47 (54.7)	39 (83.0)
	12	169	99 (58.6)	92 (92.9)
22°C	1	115	57 (49.6)	52 (91.2)
	2	128	73 (57.0)	64 (87.7)
	3	178	95 (53.4)	74 (77.9)

a Percent of survived oocytes developed to two-cell embryos.

Probability that the patterns of development are the same in all drying conditions: 4°C, P = 0.276; 22°C, P = 0.061 (Exact Fisher Test).

#### Development

The developmental stages on day 5 following ICSI (day 0 is defined as the day of ICSI) using sperm convectively dried and stored in LiCl jars at 4°C and 22°C for 1 to 12 months are listed in Table 6. All ova injected with sperm stored at 4°C and 22°C underwent some preimplantation development. The distributions of stages significantly varied with time in both the 4°C and 22°C groups (4°C, P<0.001; 22°C, P = 0.011). The rates of blastocyst formation in the 4°C group fell from 62.6% after 1 month storage to 30.4% after 12 months storage. The rates of blastocyst formation in the 22°C group fell from 28.8% after 1 month storage to 12.2% after 3 months storage. The arrests of development in the embryos that failed to develop into blastocyst varied considerably but were particularly evident at the morula stage with the exception of those stored for 3 months at 22°C. In the latter group many embryos ceased development in the pre-morula stages.

**Table 6:** The distribution of embryos at various stages of development 5 days following ICSI using sperm dried with 100 mmol/L 3-OMG for 6 minutes and stored in LiCl jars for different time at 4°C and 22°C.

		No. of embryos (%)					
Temp-erature	Storage time (mon)	2-cell	3,4-cell	5–8-cell	Compac morulae	Blastocysts	Total embryos
4°C	1	3 (3.3)	3 (3.3)	3 (3.3)	25 (27.5)	57 (62.6)	91 (100)
	2	2 (3.4)	3 (5.2)	2 (3.4)	20 (34.5)	31 (53.4)	58 (100)
	3	1 (1.9)	4 (7.5)	6 (11.3)	21 (39.6)	21 (39.6)	53 (100)
	6	3 (7.7)	6 (15.4)	2 (5.1)	15 (38.5)	13 (33.3)	39 (100)
	12	2 (2.2)	11 (12.0)	12 (13.0)	39 (42.4)	28 (30.4)	92 (100)
22°C	1	2 (3.8)	8 (15.4)	7 (13.5)	20 (38.5)	15 (28.8)	52 (100)
	2	2 (3.1)	13 (20.3)	9 (14.1)	23 (35.9)	17 (26.6)	64 (100)
	3	6 (8.1)	28 (37.8)	21 (28.4)	10 (13.5)	9 (12.2)	74 (100)

Probability that the patterns of development are the same in all drying conditions: 4°C, P<10^−5^; 22°C, P<10^−5^ (Exact Jonckheere-Terpstra Test).

**Figure 2:**
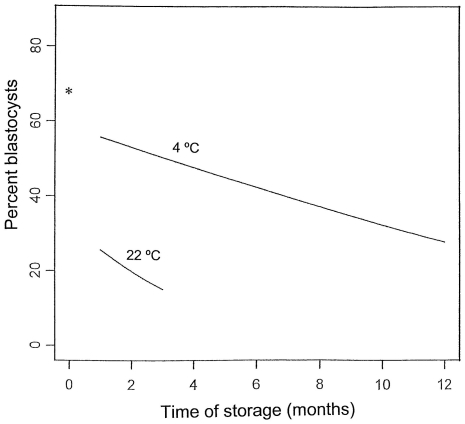
Regressions of the percent blastocyst on time of storage. Computed by transformation of the following logit responses on storage time to percentages, where p is the proportion of blastocysts: 4°C: logit(p) = −0.108 t+0.3338 22°C: logit(p) = −0.514 t−0.247.

Linear logistic regressions have been fit to the data for each of the two storage temperatures. The transformed regression lines obtained after transforming the logits to proportions are shown in Figure 2, which relates the percentage of blastocysts to the storage time at 4°C and 22°C. The 22°C line (b = −0.516, 95%, confidence limits −0.953 to −0.078) is steeper than the 4°C line (b = −0.108, 95%, confidence limits −0.160 to −0.057), showing the faster developmental failure in the 22°C group. The 4°C line is higher than the 22°C line, showing that more blastocysts were obtained when the samples were stored for the same period of time at 4°C than 22°C. The percentage of blastocysts that developed when fresh sperm was used was 68%. Extrapolation of the regression estimated from the data on storage at 4°C indicates that 10 percent of the sperm will be viable after 23.4 months or about 100 weeks.

Sperm were also convectively dried in the Na-EGTA solution containing 100 mmol/L 3-OMG for 6 min and stored in NaBr, MgCl_2_, and P_2_O_5_ jars. The percentages of blastocyst produced by sperm stored in NaBr, MgCl_2_, and P_2_O_5_ jars at 4°C for 1 month were 25.6% (10/39), 50.9% (28/55), and 25.8% (8/31), respectively. When sperm were stored in MgCl_2_ jars at 4°C for 3 month and 22°C for 1 month, the percentages of blastocysts were 15.1% (8/53) and 4.4% (2/45), respectively. No blastocyst was obtained (0/10) when the sperm stored in P_2_O_5_ jars at 22°C for 1 month were used.

### Embryo transfer and live births

**Figure 3:**
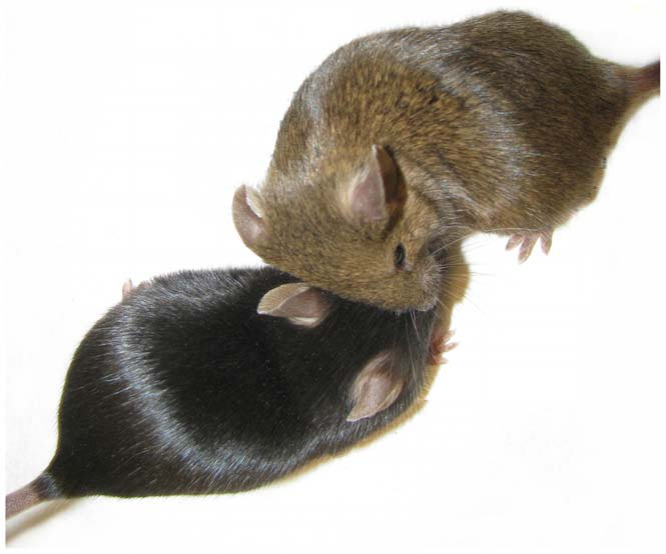
Mice produced by ICSI. Mice produced from sperm dried with 100 mmol/L 3-OMG for 6 minutes and stored in LiCl jars at 4°C for one year.

Forty-two 2- to 4-cell embryos from sperm dried in 100 mmol/L 3-OMG medium and stored in LiCl sorption jar at 4°C for 3 months were transferred into two pseudopregnant females. One female gave birth to 4 pups (4/20, 20%) and the other female had 5 pups (5/22, 23%). Thirty-eight 2- to 4-cell embryos from dried sperm stored in LiCl sorption jars at 4°C for one year and transferred into one recipient produced 2 live pups (Figure 3). From the sperm samples stored in LiCl sorption jar at 22°C for 3 months, 59 two- to four-cell embryos were transferred into three recipients to produce 2 live pups (2/59, 3%). For controls, 32 two- to four-cell embryos produced by ICSI with freshly isolated sperm were transferred into 2 females. One recipient had 4 pups (4/11, 36%) and the other had 7 pups (7/21, 30%). All the pups looked healthy at 3 weeks of age.

## Discussion

Our results show that 3-OMG protects mouse sperm from the deleterious effects of evaporative drying (Tables 4 and 6). Over thirty percent ova developed into blastocysts after being injected with spermatozoa stored for 12 months at 4°C, and 12.2% ova developed into blastocysts when injected with sperm stored for 3 months at 22°C. Although some sperm did survive desiccation in the absence of 3-OMG the yield of blastosysts was significantly less.

The use of salt sorption jars provided a constant relative humidity storage environment for dried sperm samples. This is demonstrated by the minimal changes in moisture contents of the dried sperm samples measured before and after storage. Among the four different relative humidity storage jars used, LiCl jars worked the best, as compared with NaBr, MgCl_2_, and P_2_O_5_ jars, as more blastocysts were produced from sperm stored in LiCl jars at 4°C and 22°C than the other three relative humidity storage conditions.

When dried sperm samples were vacuum-packed into a Mylar foil bag, all of the samples lost moisture during storage, at a higher rate at 22°C than 4°C [6]. When sperm samples were stored in a closed glass jar over saturated NaBr solution for 1 month, blastocyst rates were higher at both 4°C and 22°C compared to those stored in Mylar foil bags [20]. When stored in Mylar bags, the amount of water lost from the sample over time could reach a detrimental level beyond which functionality of the sperm was not preserved. This was further corroborated by the low blastocyst development rate reported here using dried sperm stored in P_2_O_5_ jars with zero relative humidity. However, it is possible that the P_2_O_5_ vapor floating in the saturated jars was absorbed by the sperm samples, impairing functionality of the sperm. These data suggest that: 1) a small amount of water in the mouse sperm is required to preserve functionality of the sperm during desiccation and storage, and 2) a stable moisture level is important for long term storage of dried sperm samples.

The entry of glucose into cells is by facilitated diffusion using members of the GLUT family of transporters. Eight of the members, GLUTs 1, 2, 3, 4, 5, 8, 9a, and 9b, have been detected in murine sperm, of which GLUTS 1, 2, 5, 8, 9b have been associated with the sperm head [21]–[23]. 3-OMG competes with glucose for uptake by the sperm, suggesting that 3-OMG and glucose share these same transport systems [24]. It has been shown that 3-OMG enters very rapidly in several types of cell [8], [9]. Thus an incubation time of 30 minutes before desiccation is more than adequate for the concentrations of 3-OMG inside and outside the cell to equilibrate. For all practical purposes the concentration of 3-OMG inside the sperm will reach 100 mmol/L before drying.

Spermatozoa are traditionally regarded as being very densely packed. Allen et al. [25], however, found that the mouse sperm nucleus contains about 64 percent water and they suggested that the organelle is not so crowded as formally believed. Three compartments are recognized in a sperm nucleus: chromatin, matrix and RNA [26]. It is the DNA that is densely packaged with protamines to form condensed chromatin [27]. A small molecule, such as trehalose, can quickly become distributed throughout a sperm nucleus (Lechene et al, unpublished), presumably associated with the nuclear matrix. It is likely that 3-OMG also becomes widely distributed in the sperm nucleus.

The molecules in all types of cell are crowded together and this fact must be taken into consideration when analyzing physiological functions [28], [29]. Removing water from spermatozoa by convective drying concentrates all the intracellular compounds, including those artificially introduced, and increases the crowding of the macromolecules. The resulting increase in viscosity is traditionally described as a passage through a series of physical states: liquid, rubbery and glassy. The effect of drying on intracellular viscosity has been quantified in pea embryos using fluorescence correlation spectroscopy and a spin probe, 3-carboxy-proxyl, which has approximately the same size as a glucose molecule [30]. As water is removed from the cells, the intracellular viscosity initially increases very slowly but after falling to a water content of 0.3 g H_2_O/g dry weight the intracellular viscosity rapidly increases. Glass typically forms when the water content falls below 0.1 g H_2_O/g dry weight. Thus, in the pea embryo, the glassy state occurs when the remaining water content is ≤0.1 g H_2_O/g dry weight; the viscous (rubbery) state occurs when 0.1<g H_2_O/g dry weight <0.3; the liquid state exists when g H_2_O/g dry weight ≥0.3.

Some spermatozoa survived convective drying without being exposed to 3-OMG; 22.5 and 10.4 percent stored for 1 and 3 months respectively at 4°C were able to fertilize mouse ova and develop into blastocysts. McGinnis et al. [6] also found that a few spermatozoa survived evaporative drying and storage for one week at room temperature without being exposed to trehalose. It is possible that the molecules in the chromatin are naturally packed sufficiently into a structure with high viscosity so that they can survive drying without additional protection. Thus the main role of a protective agent such as 3-OMG or trehalose is to protect the nuclear matrix, a function that becomes more important as storage time is prolonged.

The glass transition temperature estimates based on the phase diagram of glucose-water solutions and the final average moisture levels measured in this study suggest that the sperm do not require a glassy environment for survival and long-term stability. The glass transition temperature of 3-OMG in the storage medium used was <−40°C. Thus the sperm stored in our experiments at 4°C were not in a glassy state. The moisture content of the dried samples was in the range of 0.2–0.3 g H_2_O/g dry weight so that the sperm samples were in a rubbery state in which molecular mobility and metabolic activity are also restricted but to a lesser extent than in the glassy state. Although the exact mechanism by which convective drying affords long-term stability is not known, we believe there are several contributing factors. The functional state of the spermatozoa before drying begins may also play a role in preparing the cells for long term storage. The spermatozoa were exposed for 30 minutes before drying begins to a solution containing 3-OMG, a competitive inhibitor of glucose utilization, and EGTA, a chelator that complexes with several metallic elements having critical physiological functions. Well-known examples in mouse sperm are the Ca dependent enzymes that degrade DNA – the so-called nucleases [31], [32]. In our work, following the work of Kusakabe et al. [11], these enzymes were inhibited by chelating Ca with EGTA at a pH of 8.2–8.4 at which strong binding of the Ca occurs [33]. Also, at this pH EGTA strongly complexes with several other important elements, including Cu, Zn, Mn and Mg which are known to be essential for the biological activity of many enzymes and other bioactive proteins such as those concerned with electron transport [34], [35]. We suggest that the initial exposure of the spermatozoa to 3-OMG and EGTA before drying begins contributed to the successful storage in the rubbery state.

Mouse sperm can be preserved at above freezing temperatures for at least one year. Optimization of the humidity levels for storage with 3-OMG and other sugars may further improve the preservation of mouse sperm and sperm of other species at above freezing temperatures. Further studies on the transport of dried sperm samples are necessary to show the method may be useful in practice.
